# Enabling Open Science for Health Research: Collaborative Informatics Environment for Learning on Health Outcomes (CIELO)

**DOI:** 10.2196/jmir.6937

**Published:** 2017-07-31

**Authors:** Philip Payne, Omkar Lele, Beth Johnson, Erin Holve

**Affiliations:** ^1^ Institute for Informatics School of Medicine Washington University in St. Louis St. Louis, MO United States; ^2^ Department of Biomedical Informatics College of Medicine The Ohio State University Columbus, OH United States; ^3^ AcademyHealth Washington, DC United States; ^4^ Department of Health Care Finance Government of the District of Columbia Washington, DC United States

**Keywords:** healthcare research, information dissemination, open access to information, social networking, reproducibility of results

## Abstract

**Background:**

There is an emergent and intensive dialogue in the United States with regard to the accessibility, reproducibility, and rigor of health research. This discussion is also closely aligned with the need to identify sustainable ways to expand the national research enterprise and to generate actionable results that can be applied to improve the nation’s health. The principles and practices of *Open Science* offer a promising path to address both goals by facilitating (1) increased transparency of data and methods, which promotes research reproducibility and rigor; and (2) cumulative efficiencies wherein research tools and the output of research are combined to accelerate the delivery of new knowledge in proximal domains, thereby resulting in greater productivity and a reduction in redundant research investments.

**Objectives:**

AcademyHealth’s Electronic Data Methods (EDM) Forum implemented a proof-of-concept open science platform for health research called the Collaborative Informatics Environment for Learning on Health Outcomes (CIELO).

**Methods:**

The EDM Forum conducted a user-centered design process to elucidate important and high-level requirements for creating and sustaining an open science paradigm.

**Results:**

By implementing CIELO and engaging a variety of potential users in its public beta testing, the EDM Forum has been able to elucidate a broad range of stakeholder needs and requirements related to the use of an open science platform focused on health research in a variety of “real world” settings.

**Conclusions:**

Our initial design and development experience over the course of the CIELO project has provided the basis for a vigorous dialogue between stakeholder community members regarding the capabilities that will add the greatest value to an open science platform for the health research community. A number of important questions around user incentives, sustainability, and scalability will require further community dialogue and agreement.

## Introduction

There is an emergent and intensive national dialogue regarding the accessibility, reproducibility, and rigor of health research. This discussion is also closely aligned with the need to identify sustainable ways to expand the national research enterprise and to generate actionable results that can be applied to improve the nation’s health. The principles and practices of *Open Science* offer a promising path to address both goals by facilitating (1) increased transparency of data and methods, which promotes research reproducibility and rigor [1-4]; and (2) cumulative efficiencies wherein research tools and the output of research are combined to accelerate the delivery of new knowledge in proximal domains, thereby resulting in greater productivity and a reduction in redundant research investments [5-7]. For the purposes of the remainder of this viewpoint, we provide the following working definition for Open Science: “Open Science is the practice of science in such a way that others can collaborate and contribute and where research data, lab notes, and other research processes are freely available under terms that enable reuse, redistribution, and reproduction of the research and its underlying data and methods” [8].

Unfortunately, contradictory and sometimes conflicting positions on open science—and the way the open science paradigm might best be operationalized—demonstrate the need for greater community engagement to test the theory that open science in the health sciences can indeed improve the rigor and efficiency of research. This challenge is exemplified by the recent controversy regarding research “parasites,” [9] and the vigorous debate that ensued as a result. In response to these important and timely issues, in this viewpoint, we describe a set of lessons learned and future directions associated with an open science initiative conducted by AcademyHealth’s Electronic Data Methods (EDM) Forum, called the Collaborative Informatics Environment for Learning on Health Outcomes (CIELO) [10], targeting the broad health research community. We also highlight policy, social, cultural, and implementation-level issues, setting the stage for a vigorous community-wide dialogue concerning future activities as are needed to achieve a compelling vision of open science in health care research and all of its concomitant benefits.

## Methods

As mentioned above, we implemented a proof-of-concept open science platform for health research called CIELO [11]. Our primary goal in developing CIELO was to explore real-world information needs and end-user expectations for health research, a domain in which data provenance, privacy, security, and stewardship are of utmost importance. In pursuit of these goals, CIELO was designed and implemented based upon a set of conceptual models and functional requirements informed by systematic and rigorous user needs assessments involving representatives from the academic, private, and public sectors.

During the course of the aforementioned user-centered design process, we elucidated a number of important and high-level requirements for a research data and analytics commons. The essential requirements are as follows:

First, *a successful data and analytics commons must be able to interoperate with and leverage a variety of technologies and approaches.* There are an increasing number of technologies that can be used to enable open science, such as content management systems and standard data-centric APIs (application programming interfaces). To be successful, a commons must be able to interoperate with such technologies in a scalable and user-friendly manner.

Second, *the “app store” paradigm reflects a user experience (UX) paradigm that is comfortable and desirable for both technical and nontechnical users and can create an effective marketplace for sharing ideas across disciplines.* There exists a similarly promising body of “app store” constructs for the user-friendly submission, quality assurance, distribution, and community-wide documentation of technical artifacts, all of which can be leveraged to build an effective exchange.

Third, *social search and discovery is a critical feature to promote interaction with data and analytical tools in a data and analytics commons.* The need for social search and discovery is reflective of the primary foci of many potential commons users who seek to engage in collaborative data and analytics projects with a group of trusted and known colleagues.

In response to these preliminary user needs, CIELO was developed to provide the members of the health care research communities with *a fully functional platform and dynamic community-of-practice designed to collectively reduce time and cost of research while enhancing the reproducibility, transparency, and rigor of health research.* To achieve these aims, we implemented CIELO using a combination of the following three key features: (1) a content and version management system (such as GitHub); (2) a “folksonomy”-driven annotation and search mechanism; and (3) a simplified user experience leveraging prevailing Web application technologies. All software design and implementation activities associated with CIELO used an agile and user-centered design and evaluation process, with a specific emphasis on end-user engagement in all project phases.

**Figure 1:**
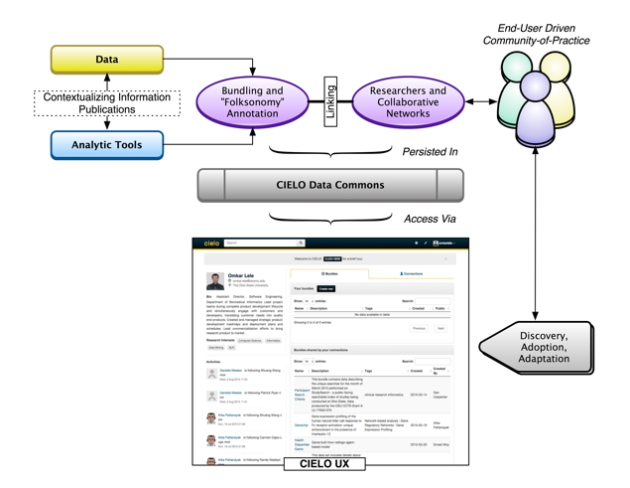
Overview of the Collaborative Informatics Environment for Learning on Health Outcomes (CIELO) architecture and workflow, emphasizing the bundling of data and analytic tools and the provisions of social search capabilities.

The resulting platform enabled the users to create analytic “bundles” (comprising both data and analytical code) to show and share their work (see Figure 1). As an early proof-of-concept to provide user feedback and demonstrate the potential impact of CIELO, we undertook a public beta release program. At the time of this submission, nearly 90 registered users from more than 20 different institutions had used CIELO.

## Results

By implementing CIELO and engaging a variety of potential users in its public beta testing, we were able to elucidate a number of additional information needs and requirements based on using an open science platform focused on health in a “real world” setting, which are as follows:

- It is important to *allow users to bundle data and code in variable ways* (eg, mapping multiple versions of code to multiple versions of data, as opposed to a one-to-one mapping of such artifacts).
- There is a need to *support multi-level sharing permissions that can evolve gracefully over the lifecycle of a project or bundle* (from private collaborative or enclaves to fully open releases of data and code).
- *Flexible and dynamic metadata management functionality* can assist in responding to the ongoing evolution of standards and requirements.
- *Cross-linkage to external data and code resources where contribution to a centralized repository is not possible,* due diverse data and code stewardship, ownership, and technical requirements, is highly desirable.
- *Support for provisioning of durable resource identifiers, such as digital object identifiers (DOIs), can increase uptake and impact.* DOIs enable attribution of work and create a value proposition for both the contribution and subsequent reuse, adaptation, and recontribution of data and analytics bundles, particularly for scholars.

## Discussion

Our initial design and development experience over the course of the CIELO project has provided the basis for a vigorous dialogue between stakeholder community members regarding the capabilities that will add the greatest value to an open science platform for the health research community. Particularly because CIELO is designed to address the needs of multidisciplinary collaborators, we believe that CIELO project provides a successful technical prototype to facilitate collaboration in health research. We have also raised a number of important questions that will require further community dialogue and agreement, as follows: How do we incentivize and sustain *participation* in these types of platforms and sharing frameworks (for example, current funding and career advancement models and metrics of scholarly success may serve as a barrier to participation)?

How do we create a sustainable *fiscal* strategy that aligns with the evolving needs of a high performing healthcare research community and the ways in which it may utilize such a commons platform?

How can we make such a platform elastic and scalable from a technical standpoint so that is can evolve gracefully over time and not become obsolete? For example, in parallel to the development of CIELO, a robust community has also arisen around the Open Science Framework (OSF) [12], which we envision as providing a complementary platform for shared data analytics workflow management and sharing of such workflows and their products. It will be important for environments such as CIELO to interoperate with those like OSF in order to create a broad-based open system “ecosystem.”

Despite these open questions, we see CIELO as a proof-of-concept for what is required to establish a functional data and analytics commons reflecting the technical and sociocultural needs of our intended end-user community. Encouraged by the robust capabilities of the platform and early user experiences, we will continue to explore the potential of CIELO by (1) identifying opportunities to deliver reference datasets within the environment that will make it even easier for individuals to share their analytics tools when source data sharing is infeasible; (2) creating incentive models to encourage the adoption and use of CIELO by a variety of stakeholders; (3) investigating novel methods to address diverse and challenging privacy and data-sharing constraints incumbent to health data in a systematic manner; and (4) continuing rigorous user-centered design processes to highlight additional functional requirements representative of end-user needs and expectations. Ultimately, we believe that projects such as CIELO represent an important effort to enable the health research community to achieve greater parity with other scientific communities, such as the natural and physical sciences, that have adopted open science paradigms and seen concomitant and exponential increases in research productivity as impact [5,6,13,14], setting the stage for achieving a compelling vision of open science and all of its concomitant benefits in the health research domain.

## Abbreviations

API: application programming interface
CIELO: Collaborative Informatics Environment for Learning on Health Outcomes
DOI: digital object identifiers
EDM: Electronic Data Methods
OSF: Open Science Framework
UX: user experience
